# Atrial Septal Aneurysm: An Incidental Finding or a Clinically Significant Anomaly?

**DOI:** 10.7759/cureus.29557

**Published:** 2022-09-25

**Authors:** Steven Imburgio, Kyle Wiseman, Ndausung Udongwo, Dhairya Gor, Dhaval Desai, Renato Apolito

**Affiliations:** 1 Internal Medicine, Jersey Shore University Medical Center, Neptune, USA; 2 Cardiology, Jersey Shore University Medical Center, Neptune, USA

**Keywords:** transesophageal echocardiography (tee), atrial septum, asa, anticoagulation, exertional dyspnea, dyspnea on exertion, atrial septal aneurysm

## Abstract

Atrial septal aneurysm (ASA) is a condition involving the bulging of the interatrial septum into one or both of the atrial chambers. We present the case of an ASA found on transesophageal echocardiogram in a patient who presented with exertional dyspnea. This case report aims to highlight the growing clinical association of ASA with arterial embolism through various mechanisms and emphasize the unknown aspects of clinical management for such patients. While there are currently no clear recommendations on whether to start anticoagulation after an ASA is diagnosed, many suggest a careful patient-centered approach for such decisions due to the reported increased risk of thromboembolic events. Further studies regarding the significance of ASA and cardioembolic events are needed.

## Introduction

An atrial septal aneurysm (ASA) is defined as a localized outpouching of the atrial septum that protrudes into the atria forming a saccular structure [1]. The exact length of the protrusion that defines an ASA varies but is typically considered an extension over 10 mm beyond the plane of the atrial septum [2]. This structural abnormality can be congenital or acquired with a suspected prevalence of around 1% to 2.5% [3,4]. ASAs were previously diagnosed during an autopsy but are now frequently detected during routine echocardiography [5]. The clinical significance, however, is not well known and the decision to anticoagulate is a point of contention. The literature has pointed to a growing association between ASAs and systemic thromboembolism, which can have significant morbidity [6]. Here we describe a case of a large ASA that was found unexpectedly during the diagnostic workup of worsening systolic heart failure. We also highlight the clinical significance of ASAs and discuss the difficult decision to anticoagulate.

## Case presentation

An 85-year-old female with a past medical history of hypertension, hyperlipidemia, second-degree heart block with dual chamber pacemaker placement, and coronary artery disease with two stents, presented with exertional paroxysmal dyspnea for the past few months. She denied any chest pain and had an otherwise negative review of systems. Physical examination at the time only revealed a grade II systolic murmur at the apex with no jugular venous distention or edema noted. Electrocardiogram was unchanged with ventricular pacing; laboratory values were within normal limits including troponin. 

**Figure 1:**
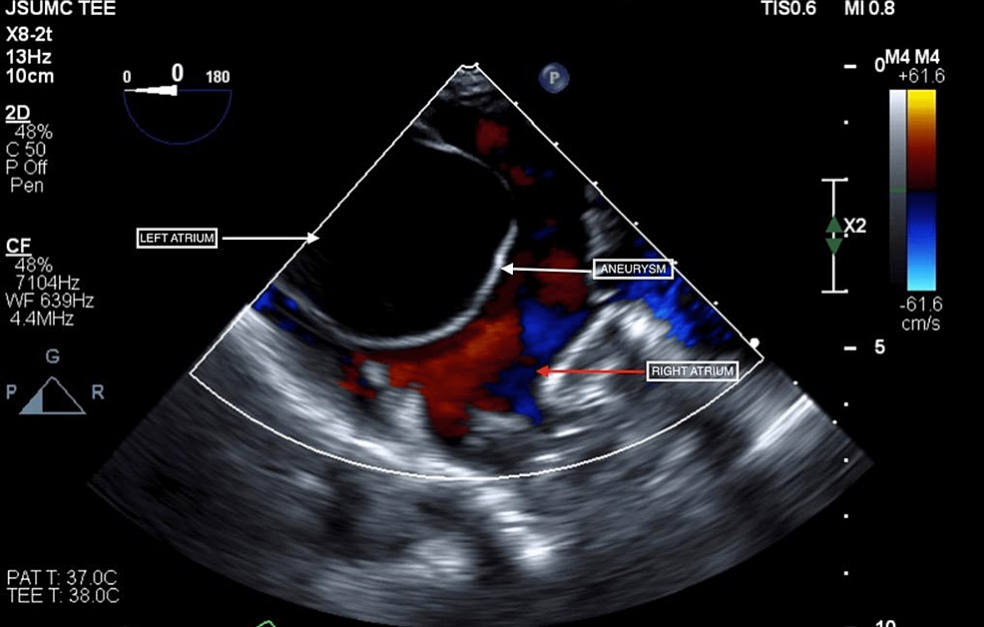
Transesophageal echocardiography (TEE) demonstrating an aneurysmal interatrial septum with fixed left to right bowing

Given the patient’s complex cardiac history, a transthoracic echocardiogram (TTE) was performed to assess the cardiac function which revealed moderate dilation of the left atrium with newly detected possible dilation of the atrial septum. The ejection fraction was reduced from 54% to 43% when compared to prior imaging results done two years prior. Due to suspicion of a structural abnormality unseen on previous echocardiograms, a follow-up transesophageal echocardiogram (TEE) was done two months later (Figure 1).

A large ASA was subsequently identified with a fixed left to right bowing, resulting in a large left atrium and impinged right atrium. However, this appeared to be an isolated finding with no intracardiac thrombus or associated atrial septal wall defect discovered. Due to prior TTEs failing to identify this large ASA in our patient, this suggested either an acquired etiology or possible limitations with TTE in successfully identifying ASAs.

After reviewing the imaging findings, there was a concern for right heart dysfunction due to a mass effect from the ASA compressing the right atrium. A right heart catheterization was performed which revealed a right atrial pressure of 10 mmHg, right ventricular pressure of 35/10 mmHg, pulmonary artery pressure of 40/20, pulmonary capillary wedge pressure of 20 mmHg, and a low cardiac output of 2.8 L/min. No further intervention was warranted, including coronary angiography due to a negative nuclear stress test. The patient was discharged home with continued treatment for new-onset systolic heart failure.

Our patient was ultimately not started on anticoagulation after her incidental diagnosis of ASA. This conclusion was reached after a careful discussion with the patient and identifying her relative lack of risk factors for systemic thromboembolic events, including lack of previous deep vein thrombosis or pulmonary embolism, along with no known history of cancer that would induce a hypercoagulable state. Additionally, we felt no urgent need to initiate anticoagulation as the imaging findings from TEE with bubble study failed to detect any intracardiac thrombus or atrial septal wall defect.

## Discussion

The patient we presented was incidentally found to have a large ASA during the workup of her exertional dyspnea. While it is uncertain whether her respiratory symptoms were a result of her structural heart defect, this case highlights the traditional route for which a large majority of ASAs are identified. Typically, this structural anomaly is discovered during a routine echocardiogram for the diagnostic evaluation of a cardiopulmonary symptom or during the workup for the etiology of a new onset stroke [5]. Our patient fell in the former category with an initial TTE only showing a dilated left atrium with suspected ballooning of the atrial septum. She required a follow-up TEE to better visualize the atrial septum and definitively confirm the diagnosis of a large ASA. Our case highlights the limitations of TTE in visualizing certain cardiac structural abnormalities such as ASAs; various case reports suggest that TEE may actually be the preferred modality to detect ASAs. For example, Tzimas and Papadopoulos presented a case where a TTE completely failed to visualize a large ASA in a patient undergoing coronary artery bypass graft (CABG) which was only discovered later by TEE [7]. To further highlight this point, a retrospective study by Mugge et al. enrolled 195 patients with known ASA and found that TEE could visualize the entire atrial septum and successfully diagnose ASA in 100% of patients, while TTE failed to identify ASA in 47% (100/195) of patients from the same group [8].

The importance of successfully diagnosing ASA stems from the fact that it has long been considered a potential source of cardiogenic embolism. One multicenter study published in the European Heart Journal found that the prevalence of cerebral ischemia and normal carotid arteries was 27.7% higher in patients with ASA as compared to the control group of patients with no documented ASA [6]. While the exact mechanism linking ASA with the increased risk of arterial embolic complications is not exactly known, one theory that has been proposed is that the presence of ASA can induce dilation of the left atrium and lead to stasis of blood flow in a manner similar to that of atrial fibrillation [5]. To date, there have been numerous case studies of patients with ASA that have revealed an atrial thrombus directly attached to the interatrial septum during imaging with an echocardiogram [9,10-12]. 

An ASA can occur as an isolated defect, as in our patient, but often will present with other concurrent structural cardiac abnormalities. A study by Giannopoulos et al. enrolled over 4500 children and found that ASA diagnosed with echocardiogram only presented as an isolated structural cardiac defect in 35.5% (17/47) of patients, while the remaining patients with confirmed ASA all had associated interatrial shunting present [3]. Several other reports support this unique relationship between ASAs with congenital heart defects including atrial septal defect, patent foramen ovale (PFO), and sinus venosus defect [4,13,14]. Furthermore, there is a strong relationship between the development of systemic thromboembolism in patients with ASA and interatrial wall defects. This is demonstrated in a multicenter study by Mugge et al. which found a high frequency of previous clinical events consistent with cardiogenic embolism in patients with ASA that were also noted to have interatrial shunts [8]. In an environment already favorable to thrombus formation from blood stasis, the right to left shunting from these interatrial defects can introduce additional blood clots directly from the deep venous system [5,8]. One study by Cheng et al. proposes that paradoxical embolism is actually the primary mechanism for stroke in patients with known ASA [4].

It is known that systemic thromboembolism is one of the major complications of ASAs which is likely due to stagnating left atrial blood flow promoting blood clot formation, combined with the often concurrent presence of interatrial shunts which provides another mechanism for introducing a thrombus into the arterial system [5,8]. The increased risk for arterial thromboembolism, paired with its potential sequela of complications such as stroke, acute mesenteric ischemia, and acute leg ischemia brings up the question as to whether there is a role for antiplatelet or anticoagulation therapy in patients with diagnosed ASA [15]. Currently, the decision to initiate medical therapy for ASA is a matter of controversy as there is a drastic lack of data in this area [16]. A recent randomized controlled study called CLOSE enrolled patients with recent cryptogenic stroke attributed to PFO associated with ASA and found that stroke recurrence was significantly less in the treatment group that underwent PFO closure with antiplatelet treatment as compared to PFO closure alone [17]. However, despite this, the data is scarce and there is no consensus on whether antiplatelet or anticoagulation provides benefits for patients with isolated ASA as in our patient.

## Conclusions

Our case aims to highlight the clinical significance and management of ASAs. Often incidentally diagnosed by the less invasive TTE, TEE is the superior imaging modality for diagnosis. The clinical significance of ASAs is tied to the question of whether there is an increased risk for systemic thromboembolic events, independent of atrial septal defects. There is currently no consensus regarding the decision to anticoagulant these patients. Thus, until further studies are completed, it is important for clinicians to utilize a shared decision approach on a case-by-case basis in consideration of the associated risks and benefits.
